# Overexpression of a Domain of Unknown Function 231-containing protein increases O-xylan acetylation and cellulose biosynthesis in *Populus*

**DOI:** 10.1186/s13068-017-0998-3

**Published:** 2017-12-27

**Authors:** Yongil Yang, Chang Geun Yoo, Kimberly A. Winkeler, Cassandra M. Collins, Maud A. W. Hinchee, Sara S. Jawdy, Lee E. Gunter, Nancy L. Engle, Yunqiao Pu, Xiaohan Yang, Timothy J. Tschaplinski, Arthur J. Ragauskas, Gerald A. Tuskan, Jin-Gui Chen

**Affiliations:** 1 BioEnergy Science Center and Biosciences Division, Oak Ridge National Laboratory, Oak Ridge, TN 37831 USA; 2 UT-ORNL Joint Institute for Biological Science, Oak Ridge National Laboratory, Oak Ridge, TN 37831 USA; 3 ArborGen Inc., Ridgeville, SC 29472 USA; 4 Department of Chemical and Biomolecular Engineering, University of Tennessee, Knoxville, TN 37996 USA; 5 Department of Forestry, Wildlife, and Fisheries, University of Tennessee, Knoxville, TN 37996 USA; 6 Center for Bioenergy Innovation, Oak Ridge National Laboratory, Oak Ridge, TN 37831 USA

**Keywords:** Acetylation, Cell wall, Cellulose, DUF231, *Populus*, Sugar release, Xylan

## Abstract

**Background:**

Domain of Unknown Function 231-containing proteins (DUF231) are plant specific and their function is largely unknown. Studies in the model plants Arabidopsis and rice suggested that some DUF231 proteins act in the process of *O*-acetyl substitution of hemicellulose and esterification of pectin. However, little is known about the function of DUF231 proteins in woody plant species.

**Results:**

This study provides evidence supporting that one member of DUF231 family proteins in the woody perennial plant *Populus deltoides* (genotype WV94), PdDUF231A, has a role in the acetylation of xylan and affects cellulose biosynthesis. A total of 52 DUF231-containing proteins were identified in the *Populus* genome. In *P. deltoides* transgenic lines overexpressing *PdDUF231A* (*OXPdDUF231A*), glucose and cellulose contents were increased. Consistent with these results, the transcript levels of cellulose biosynthesis-related genes were increased in the *OXPdDUF231A* transgenic lines. Furthermore, the relative content of total acetylated xylan was increased in the *OXPdDUF231A* transgenic lines. Enzymatic saccharification assays revealed that the rate of glucose release increased in *OXPdDUF231A* transgenic lines. Plant biomass productivity was also increased in *OXPdDUF231A* transgenic lines.

**Conclusions:**

These results suggest that PdDUF231A affects cellulose biosynthesis and plays a role in the acetylation of xylan. *PdDUF231A* is a promising target for genetic modification for biofuel production because biomass productivity and compositional quality can be simultaneously improved through overexpression.

**Electronic supplementary material:**

The online version of this article (10.1186/s13068-017-0998-3) contains supplementary material, which is available to authorized users.

## Background

The plant cell wall is important for preventing pathogen attack and structural damage from environmental perturbations and mechanical stress. Recently, plant cell walls have been highlighted as important bioenergy sources through degrading structural polymer complexes of lignocellulosic products such as cellulose, hemicellulose, pectin and lignin. Among these, pectin, lignin and hemicellulose are regarded as substrates of *O*-acetylation that impact the industrial production of biofuel and inhibit the microbial fermentation for converting sugar to ethanol by released acetate [1–3]. In particular, the acetylation of hemicellulose has been studied to a greater extent due to its relevance to biomass recalcitrance. The acetylation of xyloglucan in dicots occurs mainly on the galactosyl residues in side chains [4, 5]. In contrast, the acetylation occurs at the glucosyl residue on xyloglucan backbone in the monocot such as *Poaceae*, though such an acetylation was also found in dicot plant *Solanaceae* [6–9]. In the woody plant, the glucoronoxylan and glucomannans are mainly acetylated at the *O*-2 position and/or the *O*-3 position in xylopyranosyl or mannopyranosyl residue [3]. The acetylation at *O*-2 position of xylan has been reported to be mediated by reduced wall acetylation (RWA) in hybrid aspen [10].

In Arabidopsis, three classes of proteins including reduced wall acetylation (AtRWA), altered xyloglucan (AtAXY), and trichome birefringence (AtTBR)/TBR-LIKE (AtTBL) have been reported as modifiers of acetylation of cell wall polysaccharides. Four *AtRWA* genes have been identified and loss of function of *AtRWA* resulted in alternation of acetylation of polysaccharides. The *rwa2* single mutant reduced acetylation of pectin, xyloglucan, and xylan by up to 20% [11]. Acetylation in the quadruple loss-of-function mutant of *AtRWA* genes was reduced by 63% compared with wild type, indicating RWAs facilitate acetylation in cell wall polymers [12]. The other two protein classes of AtAXY and AtTBR/AtTBL share the conserved TBL domain and Domain of Unknown Function 231 (DUF231) [1] and are referred to as DUF231 family proteins. A total of 46 members of the DUF231 family proteins were found in the Arabidopsis genome [13]. The TBL domain has a conserved Gly-Asp-Ser (GDS) motif that can be found in esterases and lipases [14]. The DUF231 domain contains a conserved Asp-X-X-His (DXXH) motif localized toward the C-terminus following the TBL domain in most DUF231 proteins [14]. Loss of *AXY4* in Arabidopsis abolished the acetylation of xyloglucan, indicating that AXY4 functions as a xyloglucan-specific *O*-acetyltransferase [4]. AtESK1/AtTBL29, a member of AtTBL family, has been shown to transfer the acetyl residue to the 2-*O* and 3-*O* positions on xylan in vitro, and loss-of-function mutation in *ESK1*/*TBL29* rendered partial loss of 2-*O* and 3-*O*-acetylated xylan, implying that ESK1/TBL29 can function as a xylan acetyltransferase [15, 16]. It was reported that AtESK1 generates an even pattern of acetyl esters on xylan, thereby mediating the interaction of xylan with hydrophilic cellulose fibrils [17]. AtTBL3 and AtTBL31 were recently proposed to be compensators for the partial acetyltransferase activity of ESK1/TBL29 in xylan acetylation [18]. Additional AtTBL family proteins, including AtTBL32, 33, 34, and 35, have recently been reported as being mono-*O*-acetyltransferases in Arabidopsis [19, 20]. In vitro acetylation test showed that recombinant Arabidopsis TBL proteins acetylated either *O*-2 or *O*-3 mono position or 2,3-di-*O*-acetylation site [21]. In rice, a total of 66 TBL proteins were identified and, among them, OsTBL1 has been shown to function as a xylan mono-*O*-acetyltransferase [22]. Interestingly, the rice mutants of *ostbl1* and *ostbl2* were more sensitive to leaf blight pathogen, suggesting that xylan acetylation mediated by TBL plays a role in pathogen resistance [22]. Another rice GDSL motif-containing protein, brittle leaf sheath1 (BS1), was reported as the GDSL esterase for xylan deacetylation [23].

DUF231 family proteins’ activity is not limited to the acetyl transferase activity on hemicellulose. For example, the loss-of-function mutant of *TBR* and *TBL3* had increased pectin content and reduced esterification of pectin [13]. Loss of *powdery mildews resistance 5* (*PMR5*) in Arabidopsis resulted in reduction in pectin modification in cell walls together with a defect in cell expansion [24]. Through comparative genomics and amino acid sequence profiling, it was proposed that PMR5 may play a role in controlling the acylation levels of glycans via its predicted acyltransferase and esterase domain [25]. Interestingly, AtESK1 was also proposed to have similar functions as PMR5 [25]. The reduction of crystalline cellulose content was observed in the *esk1*/*tbl29* Arabidopsis mutants [15]. In addition, microarray results showed that *AtTBR* and *AtTBL3* were co-expressed with cellulose biosynthesis genes, indicating a close relationship between TBR and cellulose biosynthesis [13]. On the other hand, many Arabidopsis xylan backbone synthesis mutants have reduced cellulose content. For example, loss-of-function mutants of Arabidopsis *irregular xylem* (*IRX*) 15 and 15-L, members of DUF579 family that have been reported as biosynthetic genes related to xylan and cellulose formation have reduced cellulose content [5, 26]. Taken together, these findings suggest that DUF231 family proteins are important polysaccharide modifiers on various cell wall polymers in Arabidopsis.

So far, all functional characterizations of DUF231 family proteins have been limited to herbaceous plants, but bioinformatics analyses indicate that DUF231 proteins are also present in other species [1, 13]. In this study, we identified a total of 52 DUF231 family proteins in the woody perennial plant *Populus*. We provide characterization of one member of *Populus* DUF231 family proteins and propose that this gene is involved in both xylan *O*-acetylation and cellulose biosynthesis.

## Results

### Bioinformatics analysis of *Populus trichocarpa* DUF231 family proteins (PtDUF231)

**Fig. 1:**
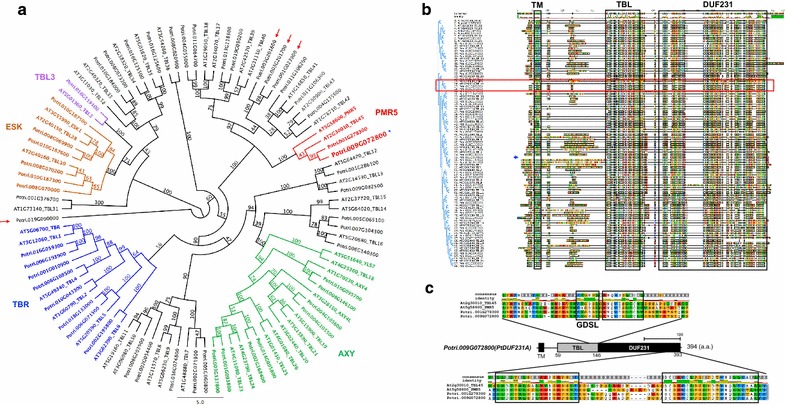
Bioinformatics analysis of DUF231-containing proteins in Arabidopsis and *Populus*. **a** Maximum likelihood phylogenetic tree of Arabidopsis and *Populus* DUF231-containing proteins. The numbers at the branches denote bootstrap confidence values. Note that 48 of the 52 PtDUF231 proteins share each node with Arabidopsis DUF231 proteins (red arrows mark four PtDUF231 proteins that are not shared a node with Arabidopsis DUF231 proteins). The location of PtDUF231A (Potri.009G072800) is indicated in the PMR5 clade by a blue asterisk. **b** Amino acid sequence alignment by MUSCLE. Note that PtDUF231A (Potri.009G072800) shows 52.5% amino acid identity with Arabidopsis PMR5 and 55.9% identity with TBL45. The closest paralog for PtDUF231A is PtDUF231B (Potri.001G278300) with 89.4% identity at the amino acid level. Blue lined brackets shown in the left illustrate the phylogenic tree as shown in **a**. Red horizontal box marks the amino acid alignment of PtDUF231A-containing node. Blue arrow points Potri.001G010900 which contains both TBL and DUF231 domains but without N-terminal sequences. Three well-conserved protein domains including TM, TBL, and DUF231 are indicated in boxes. **c** Diagram of amino acid sequence alignment of TBL and DUF231 domains among PtDUF231A, its *Populus* paralog, its Arabidopsis ortholog (PMR5) and TBL45, in the node shown in **b**. Consensus sequence was defined by 50% threshold of amino acid sequence identity. The upper panel shows sequence identity using different colors (yellow: over 50%, red: 100% conserved). All conserved regions including GDSL are indicated by the black box. Note that the TBL and DUF231 domains are highly conserved in the PdDUF231A protein

To identify DUF231-containing proteins in *Populus*, we performed a protein homolog search in the *Populus* genome (*Populus trichocarpa* v3.0 annotation) at Phytozome v11.0 web site (https://phytozome.jgi.doe.gov/pz/portal.html) using the DUF231 domain of AtTBR as a template [13]. A total of 52 *Populus* proteins were identified as DUF231-containing proteins (Additional file 1). PtDUF231 protein family members had an amino acid sequence identity of > 30% with Arabidopsis DUF231 proteins. Forty-eight of the 52 PtDUF231 proteins shared each node with Arabidopsis DUF231 proteins in the phylogenetic tree (Fig. 1a). All PtDUF231 family proteins contain a plant-specific TBL domain and a DUF231 domain (Fig. 1b). One protein, Potri.001G010900, lacks an N-terminal region, but contains both the TBL domain and the DUF231 domain (Fig. 1b). A conserved GDSL motif was identified in the TBL domain which contains approximately 50 amino acids (Fig. 1c) [14]. The TBL domain is located in proximity to the DUF231 domain in PtDUF231 proteins, similar to what was reported for Arabidopsis DUF231 (AtDUF231) proteins (Fig. 1c) [13]. As expected, the RNQWESLxCxL conserved amino acid sequences aligned next to the GDSL motif (Fig. 1c). The signature DUF231 domain motifs, LLBITxLSxxRKDGHPSxY and DCxHWCLPGxPDTWNELLYAxL, were found at the C-terminus of the proteins (Fig. 1c).

To examine how many PtDUF231 family proteins can be assigned as membrane binding proteins, as reported in Arabidopsis [1], we examined the presence of transmembrane domains (TM) in PtDUF231 proteins. Among 52 PtDUF231 proteins, 39 proteins were predicted to possess at least one TM domain at the N-terminal region (Fig. 1b; Additional file 1). Potri.010G187600 and Potri.006G140300 (with 530 and 512 amino acids, respectively; 100 more amino acids than others) were predicted to contain two TM domains (Additional file 1). In contrast, 13 PtDUF231 family proteins were predicted to not contain a TM domain (Additional file 1). The signal peptide, an indicator for transferring the protein to the endoplasmic reticulum (ER) or Golgi, was also found in nine PtDUF231 proteins, with six predicted to not contain a TM domain and three predicted to contain a single TM domain (Additional file 1).

### Expression pattern of *PdDUF231A* in different organs/tissues

No functional characterization has been reported for any member of DUF231 family proteins in *Populus*. In this study, we reported the characterization of one member of PtDUF231 family proteins, Potri.009G072800, designated as PtDUF231A. PtDUF231A clustered with the PMR5 subfamily (Fig. 1a) [24], together with its paralog encoded by Potri.001G278300 (PtDUF231B) (sharing 89.4% amino acid sequence identity with PtDUF231A). The PMR5 subfamily has been poorly characterized in plants with indications that it may function in carbohydrate modification [24, 25]. Both PtDUF231A and PtDUF231B were predicted to contain a TM domain at the N-terminus (Additional file 1).

**Fig. 2:**
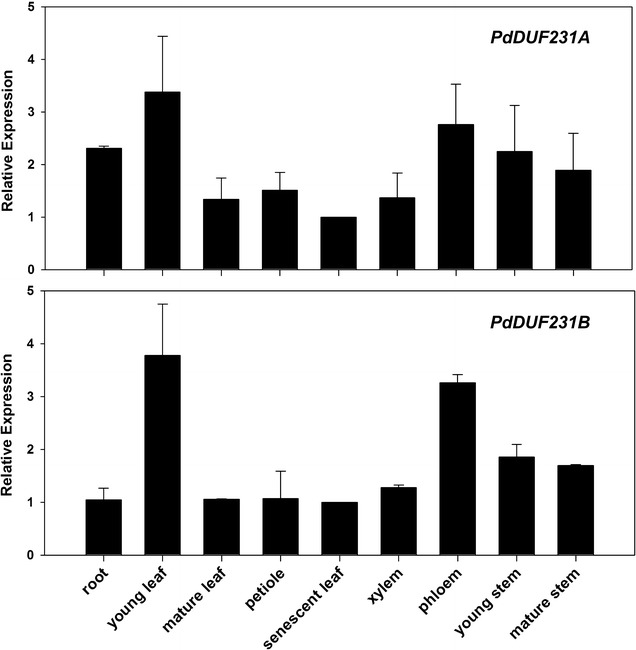
Expression pattern of *PdDUF231A* across various tissues and organs. Shown are qRT-PCR analysis using gene-specific primers for *PdDUF231A* (*Potri.009G072800*) and its paralog *PdDUF231B* (*Potri.001G278300*). The *PdUBCc* (*Populus UBIQUITIN* C) was used as an internal control. The relative expression range in each tissue/organ was determined by comparing expression level of senescent leaf (set as 1). Shown are mean values ± standard deviation (SD) of three technical replicates

As the first step toward investigating the function of *PtDUF231A*, we examined its expression pattern across various tissues and organs. We isolated RNA from various tissues and organs of *Populus deltoides* clone ‘WV94’. The full-length open reading frame of *DUF231A* gene in *P. deltoides* was designated as *PdDUF231A.* This was also the gene used for the transgenic study in the *P. deltoides* clone ‘WV94’ background described below. We designed gene-specific primers to distinguish *PdDUF231A* and *PdDUF231B* and performed a quantitative reverse transcription polymerase chain reaction (qRT-PCR) analysis. As shown in Fig. 2, *PdDUF231A* was ubiquitously expressed in all tested tissues and organs, with relatively high expression in young leaf, phloem and stem. *PdDUF231B* was similarly detected in all tested tissues and organs (Fig. 2). The only difference was that the transcript of *PdDUF231A* was higher than that of *PdDUF231B* in root (Fig. 2).

### Generation of *Populus* transgenic plants overexpressing *PdDUF231A*

**Fig. 3:**
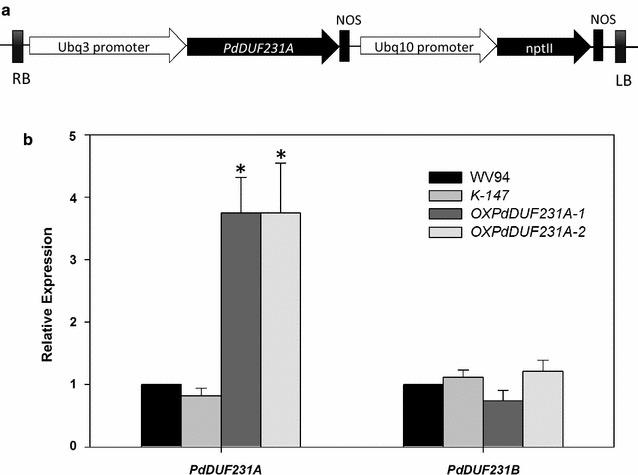
Quantitative RT-PCR analysis of the expression of *PdDUF231A* in *Populus* transgenic lines. **a** Diagram illustrating the gene construct used to generate *OXPdDUF231A* transgenic lines. **b** *PdDUF231A* expression in *OXPdDUF231A* transgenic lines. The cDNA generated from total RNA of scrapped xylem tissue was used for qRT-PCR. The *PdUBCc* (*Populus UBIQUITIN C*) was used as an internal control. Two independent *PdDUF231A* overexpression lines were examined, together with the wild-type WV94 and the empty vector control K-147. Statistical analysis was performed with three replicates in two different plants per individual transgenic line (*n* = 6). Asterisk indicates statistical significance compared to WV94 (*p* < 0.01)

To further investigate the function of *PdDUF231A*, we generated transgenic plants overexpressing *PdDUF231A* in the *P. deltoides* (genotype ‘WV94’) background. The expression of *PdDUF231A* was driven by a constitutive *UBIQUITIN3* promoter (Fig. 3a). A total of ten independent transgenic lines were generated (Additional file 2). RT-PCR analysis indicated that five among those ten transgenic lines had higher expression of *PdDUF231A* (Additional file 2). We selected two independent transgenic lines with higher *PdDUF231A* expression for further characterization and these two lines were designated as *OXPdDUF231A*-*1* and *OXPdDUF231A*-*2*. PCR analysis indicated that the copy number of the transgene was 1.8 ± 0.2 and 2.2 ± 0.4 for *OXPdDUF231A*-*1* and *OXPdDUF231A*-*2*, respectively (Additional file 3).

To quantitatively assess the *PdDUF231A* transcript level in transgenic lines, we performed qRT-PCR analysis using gene-specific primers for *PdDUF231A* and compared the transcript level of *PdDUF231A* in the transgenic plants with that in the wild-type WV94 and vector only transgenic plants (K-147). *PdDUF231B* expression was also assessed to validate that *PdDUF231A*, but not *PdDUF231B*, was overexpressed in *OXPdDUF231A.* As shown in Fig. 3, the *PdDUF231A* transcript was about fourfold higher in two transgenic lines than in WV94 and K-147, whereas the transcript level of *PdDUF231B* did not differ.

### Cellulose and glucose contents were higher in the *OXPdDUF231A* transgenic plants

**Fig. 4:**
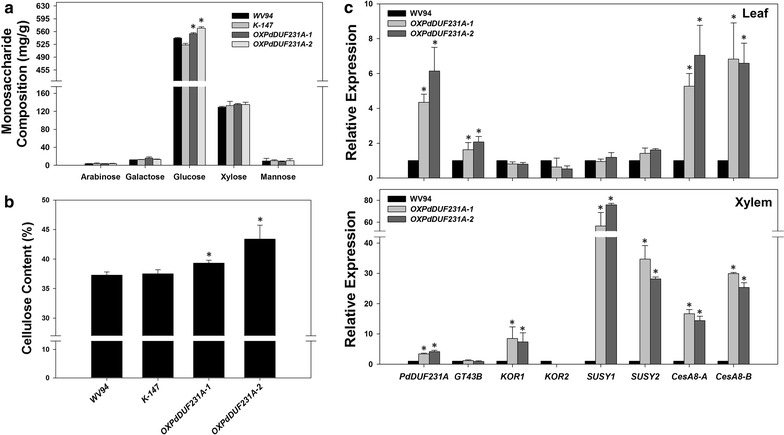
Carbohydrate and gene expression analyses in *OXPdDUF231A* transgenic lines. Two independent *OXPdDUF231A* transgenic lines (*OXPdDUF231A*-*1* and *OXPdDUF231A*-*2*), WV94 (wild type) and K-147 (empty vector control) were grown under greenhouse conditions. **a** Monosaccharide composition analysis. The monosaccharide composition was determined by ion chromatography after a two-step acid treatment. **b** Cellulose content analysis by anthrone dye staining. **c** Relative gene expression of cellulose biosynthesis-related genes in leaf and xylem. Shown are the mean value ± SD of three technical repeats of three biological replicates of leaf or xylem (*n* = 9). Asterisks indicate statistical significance compared to WV94 (*p* < 0.01)

To examine whether carbohydrate content was altered in the *OXPdDUF231A* transgenic lines, we measured monosaccharide content from stem by the NREL method [27]. The content of glucose was significantly higher in both transgenic lines than that in the control plants, whereas the contents of arabinose, galactose, xylose, and mannose were similar in all tested plants (Fig. 4a). The glucose content in *OXPdDUF231A*-*1* and -*2* was increased by 8.5 ± 4.0 and 11.4 ± 2.7% compared to WV94, respectively (Fig. 4a). To examine whether the higher glucose content observed in the *OXPdDUF231A* transgenic lines was due to higher amount of cellulose, we performed an in vitro anthrone assay to estimate the content of cellulose [28]. Both *OXPdDUF231A* transgenic lines contained significantly higher cellulose content (increased by 8–21%) than the control plants (Fig. 4b), suggesting that the higher glucose content observed in the *OXPdDUF231A* transgenic lines is likely due to higher cellulose content in the cell walls.

To seek further evidence supporting the involvement of PdDUF231A in cellulose biosynthesis, we examined the expression of several genes in the cellulose and hemicellulose biosynthesis pathways. qRT-PCR was performed using gene-specific primers for genes encoding *Populus* cellulose synthases (CesA), sucrose synthases (SUSY), and KORRIGAN (KOR) in leaf and xylem [29–31]. We also included a gene proposed to be involved in hemicellulose biosynthesis, *GT43B* [5, 32]. Among all tested genes in leaf, the most drastic changes were found for cellulose biosynthesis genes *CesA8*, whose transcript levels were four- to sixfold higher in both *OXPdDUF231A* transgenic lines than wild type (Fig. 4c). The transcript of *GT43B*, a gene encoding xylan backbone elongation factor, was also increased by approximately twofold in both *OXPdDUF231A* transgenic lines (Fig. 4c). On the other hand, the expression levels of *SUSY* and *KOR* were not significantly altered in the *OXPdDUF231A* transgenic lines (Fig. 4c). In xylem, the expression of *SUSY* family was most dramatically increased (30- to 80-fold) in both *OXPdDUF231A* transgenic plants (Fig. 4c). *CesA8* and *KOR1* were also expressed at higher levels in both *OXPdDUF231A* transgenic plants than WV94 control plant (Fig. 4c). The expression of *KOR2* and *GT43B* was not significantly altered (Fig. 4c). Collectively, we observed increased expression of genes associated with cellulose biosynthesis in *OXPdDUF231A* transgenic plants, though gene expression differences were observed between leaf and xylem tissues. These results supported that *PdDUF231A* affects cellulose biosynthesis.

### Saccharification efficiency of *OXPdDUF231A* transgenic lines

**Fig. 5:**
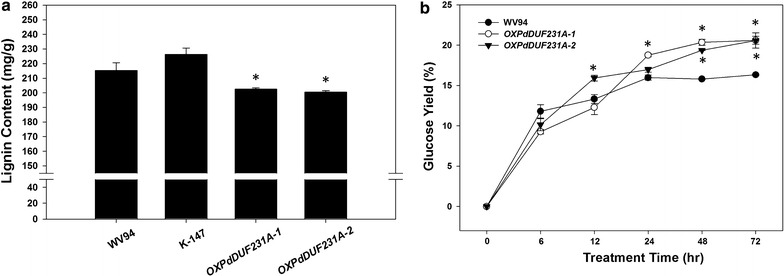
Lignin content and enzymatic saccharification assay of *OXPdDUF231A* transgenic plants. Dried *Populus* stem after debarking was subjected for lignin content measurement and saccharification analysis. **a** Lignin content of dried stem, **b** glucose yield based on total glucose content in each plant. *X* axis denotes enzymatic hydrolysis time. Each data point represents average value of two biological replicates ± SD. Asterisks indicate statistical significance compared to WV94 (*p* < 0.05)

Because PdDUF231A appeared to affect cellulose biosynthesis and contained higher content of cellulose (Fig. 4), we wanted to examine whether lignin content was altered in the *OXPdDUF231A* transgenic plants. As shown in Fig. 5a, the lignin content was reduced by 6.4–7.4% in the *OXPdDUF231A* lines compared with that in the wild type. Because both cellulose and lignin affect sugar release, subsequently we wanted to assess the enzymatic saccharification efficiency in *OXPdDUF231A* transgenic lines. We measured the amount of glucose released from enzymatic saccharification and calculated it against the total glucose content in each line. Significantly higher glucose yield in both *OXPdDUF231A* transgenic lines was observed after 48 h enzyme treatment, compared with wild-type control (Fig. 5b). At 72 h duration of enzyme digestion, the glucose yield was approximately 4% higher in *OXPdDUF231A* transgenic plants than the wild type.

### Xylan acetylation in *OXPdDUF231A* transgenic lines

**Fig. 6:**
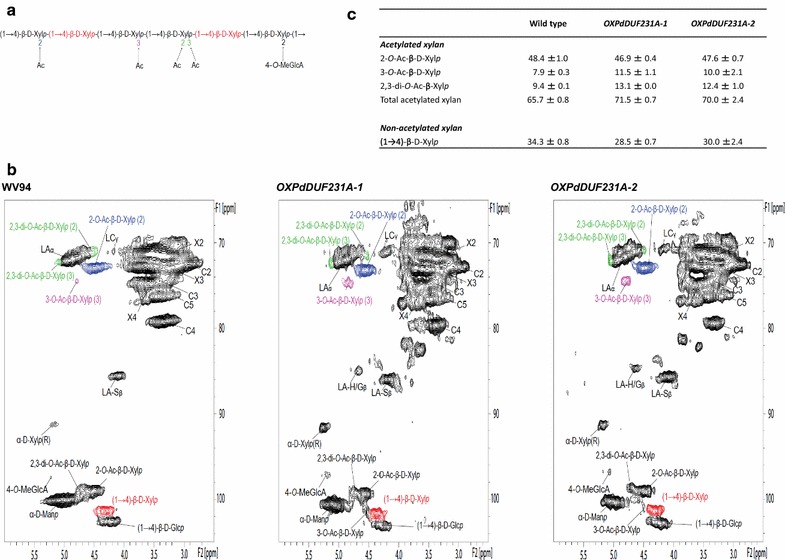
Integration analysis of xylan acetylation in *OXPdDUF231A* transgenic lines. **a** Target chemical structure detected by ^13^C-^1^H 2D HSQC NMR. **b** The NMR spectra from cell wall gels. Five different spectral types including 2-*O*-acetylated (2-*O*-AcXyl), 3-*O*-acetylated (3-*O*-AcXyl), 2,3-di-*O*-acetylated (2,3,-di-AcXyl), 4-*O*-methyl-alpha-d-glucuronic acid (4-*O*-Me-GlcA) and xylan backbone ((1-4)-β-d-Xylp) were detected in this NMR analysis. The resonance peaks of lignin were also assigned together here; *LA* β-aryl ether (β-*O*-4), *LA-H/Gβ* β-aryl ether (β-*O*-4-H/G), *LA-Sβ* β-aryl ether (β-*O*-4-S), *LC* resinol (β-β). The acetylated and non-acetylated xylan resonance peaks were used to perform integration analysis. **c** The relative integration result of acetylated groups and non-acetylated xylan. Note that 3-*O*-AcXyl and 2,3-di-*O*-AcXyl were increased in *OXPdDUF231A* transgenic lines. Shown are the mean values of two biological replicates each line ± SD

The acetyl substitution of hemicellulose, such as xyloglucan and xylan, was previously observed in the loss-of-function mutant of Arabidopsis *DUF231* genes [4, 15, 16, 18–20]. Therefore, we investigated whether acetyl groups in xylan were also affected in the *PdDUF231A* overexpression lines. We performed 2D ^1^H-^13^C HSQC NMR analysis [33] to calculate the relative acetylation levels in xylan molecules in *Populus* stems. As shown in Fig. 6a, five different types of xylan structures including 2-*O*-acetylated (2-*O*-β-d-AcXyl*p*), 3-*O*-acetylated (3-*O*-β-d-AcXyl*p*), 2,3-di-*O*-acetylated (2,3,di-*O*-Ac-β-d-Xyl*p*) xylosyl residues, 4-*O*-methyl-α-d-glucuronic acid (4-*O*-Me-GlcA), and xylan backbone [(1-4)-β-d-Xyl*p*] were observed in the NMR spectra of *OXPdDUF231A* transgenic lines and wild-type plants (Fig. 6a, b). The internal anomeric xylan correlation peak ((1-4)-β-d-Xyl*p*) appeared at 101.68/4.35 ppm, while 2-*O*-Ac-β-d-Xyl*p*, 3-*O*-Ac-β-d-Xyl*p* and 2,3-di-*O*-Ac-β-d-Xyl*p* were observed at 99.41/4.55, 101.60/4.50, and 99.26/4.71 ppm, respectively (Fig. 6b). These peaks partially overlapped, and thus the acetylated xylans were quantified with 2-*O*-Ac-β-d-Xyl*p* (C2/H2) at 73.20/4.54 ppm, 3-*O*-Ac-β-d-Xyl*p* (C3/H3) at 74.76/4.83 ppm, and 2,3-di-*O*-Ac-β-d-Xyl*p* (C2/H2) at 71.08/4.61 ppm, and compared to the xylan backbone ((1-4)-β-d-Xyl*p*) peak to obtain the relative abundance of each type of acetylated xylan. The acetyl group in each *Populus* stem was compared in two different ways. First, the total acetyl group at ~ 20.7/1.97 ppm in the cell wall samples was quantified with total xylan content based on the aforementioned assigned peaks. Since hemicellulose acetylation mostly occurs on xylan in plant cell walls [34], the observed results indirectly indicate the abundance of acetylated xylan. In addition, the relative abundance of acetyl group in *OXPdOXDUF231A* transgenic lines was confirmed by an alternate comparison using the acetylated and non-acetylated xylan peaks. The relative abundance of 2-*O*-Ac-β-d-Xyl*p* (C2/H2) was nearly the same in wild-type and *OXPdDUF231A* lines, whereas those of 3-*O*-Ac-β-d-Xyl*p* (C3/H3) increased from 7.9% in wild-type to 10.0–11.5% in *OXPdDUF231A* transgenic lines (Fig. 6c). The 2,3-di-*O*-Ac-β-d-Xyl*p* (C2/H2) was increased from 9.4% in the wild-type to 12.4–13.1% in the transgenic plants (Fig. 6c). The total acetylated xylan was increased from 65.7% in wild-type to 70–71.5% in *OXPdDUF231A* transgenic plants (Fig. 6c). The 4-*O*-methyl-α-d-glucuronic acid (MeGlcA) substitution reported in a previous study [35] was only barely observed in this NMR analysis. These results indicated that acetylation of xylan was influenced by overexpression of *PdDUF231A*.

### Biomass production in *OXPdDUF231A* transgenic lines

**Fig. 7:**
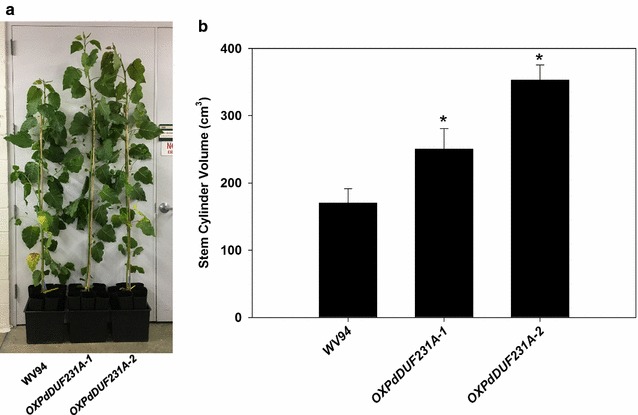
Stem volume of *OXPdDUF231A* transgenic plants. **a** Whole plant images of 6-month-old *OXPdDUF231A* plants grown under greenhouse conditions (bar = 30 cm). **b** Comparison of estimated stem volume between *OXPdDUF231A* transgenic plants and WV94 control. The stem volume was estimated by using the *πr* ^2^ *h* equation with height and diameter of primary stem. Shown are the average values of calculated stem cylinder volumes ± SD (*n* = 3)

We observed that *OXPdDUF231A* transgenic lines were larger than control plants under our greenhouse conditions. Therefore, we measured the diameter and height and used the stem volume to estimate the biomass amount of *OXPdDUF231A* plants and compared it with the WV94 control plants. As shown in Fig. 7, the stem volumes of both *OXPdDUF231A* transgenic plants were significantly higher than those of the control plant, suggesting that overexpression of *PdDUF231A* increases biomass production.

### Metabolite profiles of *OXPdDUF231A* transgenic lines

To seek further evidence supporting a role of PdDUF231A in modifying cell wall chemistry, we analyzed the metabolite profiles of *OXPdDUF231A* transgenic lines. We found that overexpression of *PdDUF231* had greatly altered the leaf metabolite profiles relative to that observed for the empty vector control plants (Tables 1, 2; Additional file 4). The greatest upregulated metabolite that was statistically significant (*p* ≤ 0.05) was a 30.75-fold increase for a partially identified metabolite (13.27 235 xylopyranoside) that is likely an aromatic glycoside. With the two major *m/z* being 204 and 235 and the metabolite eluting earlier than known glucosides, the metabolite was tentatively identified to be a xylopyranoside conjugated to an aromatic moiety, possibly coniferyl alcohol, which would generate the observed *m/z* 235 if conjugated on the alcohol rather than on the aromatic ring as it is for coniferin. Additionally, other major upregulated aromatic metabolites included 1,5-dicaffeoyl-shikimate (25.92-fold), 1,2,3-benzenetriol (pyrogallol) (5.42-fold) and salicyl alcohol (3.38-fold). Several organic acid and fatty acid metabolites were also upregulated, including maleic acid (3.49-fold), erythronic acid (3.46-fold), phytol (2.31-fold), digalactosylglycerol (2.12-fold) and linoleic acid (1.27-fold). In contrast to the relatively small number of upregulated metabolites, there was a large number of downregulated metabolites including amino acids, organic acids, and flavonoids. Significantly downregulated amino acids included lysine, asparagine, ornithine (includes that generated from arginine breakdown), glutamine, 5-oxo-proline, threonine, alanine, phenylalanine, glutamic acid, aspartic acid, and serine, which ranged from 0.03- to 0.40-fold of that observed in control plants. Downregulated organic acids included oxalomalic acid, α-keto-glutaric acid, citraconic acid, citric acid, and succinic acid, which were reduced from 0.08- to 0.63-fold of the controls. Flavonoids that were reduced included rutin, luteolin, unknown 17.80 501 559 471 flavonoid, quercetin, and kaempferol that were reduced to 0.04- to 0.12-fold of that of controls. Although most higher-order salicylates were unchanged, those that are conjugated to hydroxycinnamates, including populosides B and C, were reduced to 0.21- and 0.41-fold, respectively. Similarly, many partially identified aromatics conjugated to hydroxycinnamates, including *p*-coumaric acid, caffeic acid, and ferulic acid were also greatly reduced in leaves of plants overexpressing *PdDUF231A.* These metabolites have characteristic *m/z* of 219, 307, and 249, respectively, when conjugated as esters. An exception was 1,5-dicaffeoyl-shikimate which was elevated almost 26-fold, but coupled with a decline in shikimic acid by 0.34-fold. Together, these results indicate major shifts in major aromatic pathways with restricted production of flavonoids and most hydroxycinnamate conjugates.

**Table 1:** List of metabolites with increased content (µg/g FW sorbitol equivalents) in leaf tissues of 6-month-old greenhouse-grown *OXPdDUF231A* versus K-147 plants

Metabolite [retention time (min); key *m/z*]	*OXPdDUF231A*/K-147		*OXPdDUF231A*		K-147	
	Fold change	*p* value	Avg	sem	Avg	sem
19.63 171 Feruloyl-caffeoyl glycoside	#DIV/0!	0.282	0.4	0.3	0	0
17.04 354 Guaiacyl lignan	#DIV/0!	0.094	1.0	0.3	0	0
17.33 354 Syringyl lignan glycoside	#DIV/0!	0.081	1.2	0.4	0	0
18.48 Feruloyl-caffeoyl-shikimate glycoside	59.16	0.083	15.2	5.0	0.3	0.0
13.27 235 Xylopyranoside	30.75	0.001	2685.9	330.7	87.3	15.0
1,5-Dicaffeoyl-shikimate	25.92	0.008	99.0	17.9	3.8	0.3
21.01 557 193 647 Glycoside	15.61	0.252	4.5	2.3	0.3	0.0
13.63 278 309 227	11.67	0.002	7.6	1.0	0.7	0.0
18.43 616 386 Caffeoyl-shikimate glycoside conjugate	9.61	0.243	0.8	0.4	0.1	0.0
Syringin	9.06	0.199	13.7	5.9	1.5	0.1
1,2,3-Benzenetriol	5.42	0.002	15.2	1.8	2.8	0.5
Raffinose	4.75	0.066	86.6	21.4	18.2	4.0
Maleic acid	3.49	0.028	406.5	69.4	116.5	38.4
Erythronic acid	3.46	0.046	42.4	8.5	12.2	1.0
18.11 235 Phenolic glycoside	3.46	0.263	0.6	0.2	0.2	0.0
Salicyl alcohol	3.38	0.020	113.5	18.1	33.5	5.3
Galactinol	3.25	0.160	74.7	22.4	23	4.1
12.81 539 359 320	3.23	0.026	15.1	2.5	4.7	0.3

Metabolites were analyzed as trimethylsilyl derivatives by gas chromatography–mass spectrometry. Data are the average (avg) and standard error of the mean (sem) of six *OXPdDUF231A* plants (three plants from each of two independent transgenic lines) and three K-147 control plants. Unknown and partially identified metabolites are designated by retention time, key mass-to-charge (*m/z*) ratios, and identified moieties. #DIV/0! represents an integration area divided by zero (absence)

**Table 2:** List of metabolites with decreased content (µg/g FW sorbitol equivalents) in leaf tissues of 6-month-old greenhouse-grown *OXPdDUF231A* versus K-147 plants

Metabolite [retention time (min); key *m/z*]	*OXPdDUF231A/K*-*147*		*OXPdDUF231A*		*K*-*147*	
	Fold change	*p* value	Avg	sem	Avg	sem
Iminodiacetic acid	0.02	0.003	3.3	0.9	188.2	63.6
Asparagine	0.03	0.000	2.4	1.0	68.5	14.7
10.84 158 200 302	0.03	0.000	0.6	0.1	21.2	2.2
Lysine	0.03	0.003	2.3	1.0	90.7	29.9
Ornithine	0.04	0.000	50.1	35.3	1290.1	170.1
Rutin	0.04	0.000	0.5	0.4	12.8	2.6
Luteolin	0.05	0.004	0.6	0.1	11.4	3.9
17.80 501 559 471 Flavonoid	0.06	0.005	1.4	0.3	21.2	7.4
7.92 218 231	0.06	0.000	0.3	0.1	4.7	0.6
13.75 219 Coumaroyl glycoside	0.07	0.001	1.3	0.1	17.6	4.3
9.49 302 316 288 242 208	0.07	0.000	4.5	1.7	68.5	11.6
α-Keto-glutaric acid	0.08	0.000	9.7	2.3	114.7	14.5
1,6-Anhydroglucose	0.08	0.000	16.7	3.0	209.7	37.7
Oxalomalic acid	0.08	0.000	7.5	1.3	96.6	17.6
Glutamine	0.08	0.001	6.8	1.8	90.3	24.4
14.11 521 171 289 Glycoside	0.09	0.013	1.9	0.7	20.0	8.2
3-Amino-2-piperidone	0.09	0.007	3.7	3.1	39.8	12.9
10.58 315 330 200 172	0.09	0.000	1.3	0.9	14.3	2.0
10.15 368 353 271 242 184	0.09	0.000	6.4	2.4	71.1	10.9
8.99 242 257 99	0.10	0.003	2.2	0.7	22.0	6.7

Metabolites were analyzed as trimethylsilyl derivatives by gas chromatography–mass spectrometry. Data are the average (avg) and standard error of the mean (sem) of six *OXPdDUF231A* plants (three plants from each of two independent transgenic lines) and three K-147 control plants. Unknown and partially identified metabolites are designated by retention time, key mass-to-charge (*m/z*) ratios, and identified moieties

## Discussion

In this study, we identified a total of 52 DUF231-containing proteins in *Populus* (Fig. 1) and characterized one member of this protein family, PdDUF231A. PtDUF231A was clustered with the PMR5 subfamily in the phylogenetic tree (Fig. 1a). The PMR5 subfamily has been poorly characterized in plants with indications that it may function in carbohydrate modification [24, 25]. Overexpression of *PdDUF231A* resulted in increases in cellulose content, sugar release, and 3-*O*-acetylated xylan and 2,3-*O*-acetylated xylan (Figs. 4, 5, 6), suggesting that PdDUF231A plays a role in both xylan acetylation and cellulose biosynthesis. The phenotype of increased 3-*O*-acetylation on xylan in *Populus* transgenic plants overexpressing *PdDUF231A* is opposite to that reported in loss-of-function mutants of *DUF231* genes in Arabidopsis [17, 18], suggesting that a common function of xylan acetylation by *DUF231* genes may exist in both herbaceous and woody species. The increased xylan acetylation which would limit xylan chain elongation may have driven the large accumulation of the partially identified xylopyranoside aromatic metabolite eluting at 13.27 min with a key *m/z* 235 (Table 1).

### PdDUF231A and cellulose biosynthesis

Cellulose forms the largest portion of secondary cell walls. For biofuel conversion and production using plant biomass, the availability and utilization of cellulose is critical. Increases in cellulose and glucose contents were observed in two independent *Populus* transgenic lines overexpressing *PdDUF231A* (Fig. 4). In Arabidopsis, reduction in cellulose content has been observed in loss-of-function mutants of *DUF231* genes, such as *esk1* and *tbr* [13, 16], suggesting that involvement in cellulose biosynthesis may be another common feature of *DUF231* genes in herbaceous and woody species. Given that the other major cell wall monosaccharides were not negatively impacted, the bulk of increased carbon partitioning to glucose and cellulose in plants overexpressing *PdDUF231A* likely occurred at the expense of soluble flavonoids and hydroxycinnamate conjugates as indicated in the metabolite profiles (Tables 1, 2).

Co-expression analysis based on microarray results showed that *TBR* (At5G06700) and *TBL3* (At5G01360) are co-expressed with cellulose biosynthesis genes, although whether the expression of cellulose synthase genes is affected by the modification of *DUF231* gene expression has not been tested. Here, we showed that overexpression of *PdDUF231A* resulted in increase in the expression of *SUSY* and *CesA8* and increase of cellulose and glucose contents (Fig. 4), reinforcing the view of close relationship between DUF231 proteins and cellulose biosynthesis. To date, CesA4, CesA7, and CesA8 have been reported to be involved in the assembly of the CesA complex responsible for secondary cell wall formation [36, 37]. SUSY also participates in cellulose biosynthesis by producing UDP-glucose to elongate cellulose fibril. *Populus* transgenic plants heterologously expressing the cotton (*Gossypium hirsutum* L.) *SUSY* gene had elevated cellulose content [38]. Reduction of hybrid aspen (*Populus tremula* L. × *tremuloides Michx.*) *SUSY* resulted in a decrease in wood density together with reduced contents of lignin, hemicellulose, and cellulose [39]. In addition, the transgenic tobacco expressing *P. simonii* × *P. nigra* SUSY2, a protein highly similar to *Populus trichocarpa* SUSY2, showed increased cellulose content and fiber length [40]. *SUSY* gene expression was most drastically elevated in the xylem of *OXPdDUF231A* transgenic plants, supporting the view that PdDUF231A is involved in cellulose biosynthesis (Fig. 4). Given that PdDUF231A does not appear to be a transcription factor (i.e., without a DNA binding motif), its influence on *SUSY* and *CesA8* expression is likely an indirect effect.

### PdDUF231A and xylan biosynthesis

It should be noted that the expression of *GT43B,* a putative marker gene for xylan biosynthesis, was slightly upregulated in the leaf, but was not altered in the xylem of *OXPdDUF231A* transgenic plants (Fig. 4). Carbohydrate composition analysis did not indicate alteration in xylose content in the stem samples (Fig. 4). Characterization of xylan-deficient mutants *irx9*, *irx10,* and *irx10*-*like* suggested that *GT43* and *GT47* are involved in xylan elongation, and their xylan synthase activity has been demonstrated experimentally [32, 41, 42]. Although we cannot rule out a possible role of PdDUF231A in xylan biosynthesis, given the slight increase of *GT43B* expression in the leaf of *OXPdDUF231A* transgenic plants, such a role may not be major since the expression of *GT43B* in the xylem was not altered in *PdDUF231A* overexpression lines (Fig. 4).

### PdDUF231A and xylan acetylation

Although PdDUF231A may have a minor role in xylan biosynthesis, it potentially has an important role in the modification of xylan. The 2D-HSQC NMR analysis showed an increase of *O*-acetylated xylan in *OXPdDUF231A* transgenic lines (Fig. 6), suggesting that PdDUF231A is involved in the acetylation of xylan. More specifically, acetylations at 3-*O*- and 2,3,-di-*O*-xylosyl residues on xylan were increased in both *OXPdDUF231A* transgenic lines (Fig. 6), indicating that PdDUF231A may specifically regulate these two types of acetylation. Acetylation at 3-*O*-xylosyl residue by PdDUF231A is consistent with the studies on DUF231 proteins in Arabidopsis [18–20]. It should be noted that the acetylation at 2,3-di-*O*-xylan was also increased in the *OXPdDUF231A* transgenic lines, but we could not specify whether this increase was induced by another acetylation of either mono-acetylated xylan or by simultaneous acetylation at 2- and 3-xylosyl residues on xylan. As a xylan-specific acetyltransferase among AtDUF231 family protein, *AtESK1* mutant has a drastic reduction of 2-*O*-acetylated xylan [16]. However, mono 2-*O*-acetylated xylan was not drastically altered in *OXPdDUF231A* (Fig. 6), implying the acetylation at 3-*O*-xylosyl residue on xylan was not compensated by reduction of 2-*O*-AcXyl*p* in *P. deltoides*. Additionally, because AtESK1 has recently been shown to be necessary for generating the even pattern of acetyl esters on xylan which is required for normal interaction with cellulose fibrils [17] and *OXPdDUF231A* transgenic lines showed increased glucose release (Fig. 5), it remains unknown whether excess xylan acetylations (i.e., via *PdDUF231A* overexpression in the present study) may have made cellulose fibrils more accessible for digestion by enzymes.

Although in the present study, we present evidence supporting the association of PdDUF231A with cellulose biosynthesis and xylan acetylation, the biochemical activity of PdUDF231A remains to be determined. We cannot rule out the possibility that PdDUF231A may also have a role in the modification of other cell wall polysaccharides. The specific mechanism underlying increased acetylation of xylan and increased cellulose content in *PdDUF231A* overexpression lines remains unknown. However, because reduced xylan acetylation and reduced cellulose content were observed in loss-of-function mutants of *AtDUF231* in Arabidopsis, the association of xylan acetylation and cellulose biosynthesis may represent a general feature of action of DUF231 proteins. A precise mechanism of such correlations is worth further investigation and may have profound impact on the conversion of plant biomass for biofuel production. In addition, reduced lignin content was observed in the *Populus* transgenic lines overexpressing *PdDUF231A*. It is unknown whether this is an indirect effect due to increased cellulose biosynthesis. Finally, increased sugar release was observed in *PdDUF231A* overexpression lines. How increased cellulose content, reduced lignin content, and increased xylan acetylation were playing out in the process of enzymatic saccharification is an interesting topic that is worth further investigation.

## Conclusions

PdDUF231A enhances both cellulose biosynthesis and xylan acetylation, coupled with large-scale shifts in carbon partitioning away from flavonoids and many hydroxycinnamate conjugates. One important feature of *PdDUF231A* overexpression lines is that both the saccharification efficiency and biomass production were increased. This makes *PdDUF231A* an attractive target for genetic modification through overexpression for biofuel conversion and production.

## Methods

### Protein amino acid sequence analysis and phylogenetic analysis

To identify DUF231-containing proteins encoded by the *Populus* genome, we used the amino acid sequence of the DUF231 domain (from amino acid 429 to amino acid 590) of the AtTBR (AT5G06700) protein as a query to search the *Populus trichocarpa* v3.0 genome annotation database through a BLAST search by TBLASTN (v. 2.2.26) using the BLOSUM62 database integrated in Phytozome v11.0 (https://phytozome.jgi.doe.gov). In a second search, we used the full-length amino acid sequence of Potri.001G010900, the PtDUF231 family protein showing the highest amino acid sequence identity (61.7%) with AtTBR, as a query. The proteins with short amino acid length (< 300 A.A.) or low amino acid sequence identity (< 30%) with the DUF231 domain of AtTBR were filtered out of the protein alignment and phylogenetic analyses.

The Arabidopsis DUF231-containing proteins were adopted from the published study [13]. Complied full-length PtDUF231 and AtDUF231 proteins were aligned using MUSCLE [43] integrated in Geneious software (v8.1.2; Biomatters Ltd., New Zealand). For phylogenetic analysis, amino acid alignments were subjected to the PhyML 3.0 [44]. The phylogenetic tree was constructed by LG matrix for protein substitution modeling with bootstrap resampling using 1000 replicates. To predict the TM domain, the full-length amino acid sequences of PtDUF231 proteins were subjected to the TMHMM Web-based software (v2.0) (www.cbs.dtu.dk/servies/TMHMM) [45]. Significant TM predictions were determined by selecting the probability score over 0.8. To assess the probability of signal peptides, the same amino acid sequences were subjected to SignalP v4.1 server (http://www.cbs.dtu.dk/services/SignalP) under a valuable signal sequence selection score over 0.5 [46].

### Plant materials and biomass measurement

The full-length open reading frame of *PdDUF231A* was amplified from *P. deltoides* clone ‘WV94’, cloned into the binary vector and used in *Agrobacterium*-mediated transformation at ArborGen LLC, Ridgeville (SC), as described previously [47, 48]. A total of ten independent transgenic lines were generated. Transgenic plants including the empty vector transformed control plants and wild type (WV94) were grown in the greenhouse at Oak Ridge National Laboratory at constant 25 °C and 16-h day length.

To estimate stem volume, we measured stem diameter at a position that was 1 cm above the base of the primary stem and measured the total height from the base of the primary stem to the apical top. By using these parameters, we estimated the stem volume using the *v* = *πr* ^2^ *h* equation (*v*: volume, *r*: diameter, *h*: height).

### RT-PCR and qRT-PCR analyses

For the expression analysis of *PdDUF231A* expression in different tissues/organs, total RNA was prepared from root, young leaf, mature leaf, young stem (internodes 1–3), mature stem (internodes 6–8), petiole of mature leaf, phloem (bark of mature stem), and xylem (scrapped stem under bark of mature stem) [49]. Total RNA extraction and qRT-PCR were performed by the same method as described previously [48].

For RT-PCR analysis for transgenic line selection, the PCR was performed with dreamTaq enzyme solution with 1 µL of two times diluted cDNA (Thermo Fisher Scientific). PCR were performed as follows: denaturation at 95 °C for 2 min followed by 30 cycles of 95 °C for 30 s, 56 °C for 30 s and 72 °C for 20 s. The final extension reaction was performed at 72 °C for 7 min. As an internal control, we used *PdUBCc* gene in the same manner as above, but replaced the 28 cycles with an annealing temperature of 57 °C in the PCR. The gene-specific primers used and their sequences are listed in Additional file 5.

### Gene copy number quantification in transgenic plants

To determine the copy number of *PdDUF231A* transgene in the transgenic lines compared to WV94, genomic DNA of *PdDUF231A* gene was quantified by quantitative PCR [50]. Genomic DNA was extracted from mature leaf using a DNeasy Plant Mini kit (Qiagen, Heiden, Germany). One hundred ng of genomic DNA was amplified with *PdDUF231A*-specific primes as described in “RT-PCR and qRT-PCR analyses”. *PdUBCc* was used for internal control. The relative transgene quantification was determined by the 2^−ΔΔ*Ct*^ equation [51].

### Cell wall chemical composition analysis

Two-step sulfuric acid (H_2_SO_4_) hydrolysis with the extractives-free biomass to analyze carbohydrate contents in the air-dried stem was performed as described previously [48]. The extractive-free stem was prepared by ethanol/toluene (1:2, v/v) extraction followed by hydrolyzing with 72% H_2_SO_4_ at 30 °C for 1 h. The mixture was diluted to 4% concentration of H_2_SO_4_, and then more hydroxylation performed at 121 °C using an autoclave for 1 h. The hydrolysate and residual solids after two-step acid hydrolysis were separated by filtration. The filtered liquid fraction was used for sugar composition analysis using a Dionex ICS-3000 ion chromatography system.

To measure lignin content, we collected separately acid-soluble and -insoluble fraction from hydrolysate and solid residue. Acid-soluble lignin content was measured at 240 nm with UV/Vis spectroscopy. The lignin content in the acid-insoluble fraction was determined using solid pellet after filtration by the NREL protocol [27].

### Anthrone assay

To determine glucose content using colorimetric measurement with anthrone dye, we used a total of 15 mg of milled dried stems of 6-month-old *Populus* plants. Sample preparation and anthrone binding assay have been described previously [48]. A total of 15 mg of milled dried stem of *Populus* transgenic plants and WV94 control plants (6-month-old grown in greenhouse) were dissolved in 500 µL of acetic nitric acid reagent [1:8:2 (v/v) of nitric acid:acetic acid:water] (Sigma-Aldrich, St. Louis, MO) followed by heating at 98 °C for 30 min. The undissolved pellet was collected by centrifugation for 10 min at 14,000 rpm. The pellet was dissolved in 600 μL of 67% sulfuric acid for 1 h at room temperature. The dissolved solvent phase was separated from the pellet by centrifugation for 5 min at 14,000 rpm. Twenty μL of solution was diluted to ten times with deionized water. The diluted solution was diluted again to five times and then mixed with freshly prepared anthrone solution (0.5 mg of anthrone/mL of concentrated sulfuric acid) (Sigma-Aldrich, St. Louis, MO). The anthrone and sample mixture was boiled at 96 °C for 10 min and cooled down at 4 °C. The glucose content was determined by measuring the absorbance at 630 nm wavelength compared to glucose standard solution. Based on the measurement of glucose content, the cellulose content (%) was converted by applying the equation of [(glucose quantity × 600 (dilution factor)]/[15(initial sample amount) × 1000)] × 100.

### Two-dimensional heteronuclear single quantum coherence nuclear magnetic resonance (2D-HSQC NMR) analysis

Two biological replicates of each line were used for 2D-HSQC NMR analysis. *Populus* stems were ground with a Wiley mill and extracted with ethanol:toluene (1:2, v:v) for 24 h. The extractives-free samples were air dried at ambient temperature and ground using a planetary ball mill (Retsch PM 100) spinning at 580 rpm with zirconium dioxide (ZrO_2_) vessels (50 mL) containing ZrO_2_ ball bearings (10 mm × 10) for 2 h and 30 min (5 min grinding and 5 min break) for whole cell wall NMR analysis [33]. The ball-milled, whole cell wall sample (100–130 mg) was loaded in a 5 mm NMR tube with DMSO-*d*_6_/HMPA-*d*_18_ (4:1, v:v, ~ 0.5 mL). NMR spectra were acquired at 298 K using a Bruker Advance III 400-MHz spectroscopy equipped with a 5-mm Broadband Observe probe (5-mm BBO 400 MHz W1 with Z-gradient probe, Bruker). Two-dimensional (2D) ^1^H-^13^C heteronuclear single quantum coherence (HSQC) experiment was performed using a Bruker standard pulse sequence (‘hsqcetgpsi2’) with the following parameters: spectral width of 11 ppm in F2 (^1^H) with 2048 data points and 190 ppm in F1 (^13^C) with 256 data points; 128 scans (NS) and 1 s interscan delay (D1). Volume integration of contours in HSQC spectra was carried out using Bruker’s TopSpin 2.1 software. Assignments of peaks from NMR spectra were based on previous publications [52, 53]. For comparing the relative content of acetyl group in xylan, non-acetylated (1 → 4)-β-d-Xyl*p* and acetylated ones including 2-*O*-acetylated (2-*O*-Ac-β-d-Xyl*p)*, 3-*O*-acetylated (3-*O*-Ac-β-d-Xyl*p*), and 2,3,-di-*O*-acetylated (2,3-di-*O*-Ac-β-d-Xyl*p*) xylosyl residues in 2D ^1^H-^13^C HSQC NMR spectra were integrated.

### Enzymatic saccharification assay

Air-dried *Populus* stem of 6-month-old after peeling was Wiley-milled with 40 mesh. The methods for enzyme treatment and sugar detection have been described previously [48]. The enzymatic saccharification assay was performed without any pretreatment process (i.e., without strong acid solution treatment). For each sample, 250 mg of dried sample was dissolved in 50 mM citrate buffer (pH 4.8) complemented with Novozymes CTec2 (70 mg of enzyme/gram of biomass) and then incubated at 50°C with 200 rpm shaking. The time course samples were collected at 0, 6, 12, 24, 48, and 72 h after incubation. The enzyme was deactivated by boiling water before carbohydrate measurement. Ion chromatography was performed to measure the released sugar with Dionex ICS-3000 ion chromatography system. The measurement value displayed the average value of two biological replicates.

### Metabolite profiling by gas chromatography–mass spectrometry

Leaves (LPI 5) of ~ 9-month-old transgenic *OXPdDUF231A* (DUF231A) (*n* = 6; 3 plants from each of two independent transgenic lines) and empty vector control (K-147) *P. deltoides* ‘WV94’ plants (*n* = 3) growing in the greenhouse were fast frozen in liquid nitrogen and stored at − 80 °C. The leaf tissues were ground with liquid nitrogen in a chilled mortar and pestle with ~ 50 mg FW of leaf tissue, and were subsequently twice extracted with 2.5 mL 80% ethanol overnight and then combined prior to drying a 1.0 mL aliquot in a nitrogen stream. Sorbitol was added before extraction as an internal standard to correct for differences in extraction efficiency, subsequent differences in derivatization efficiency, and changes in sample volume during heating. Dried extracts were dissolved in 500 μL of silylation–grade acetonitrile, followed by the addition of 500 μL *N*-methyl-*N*-trimethylsilyltrifluoroacetamide (MSTFA) with 1% trimethylchlorosilane (TMCS) (Thermo Scientific, Bellefonte, PA), and samples then heated for 1-h at 70 °C to generate trimethylsilyl (TMS) derivative [54, 55]. After 2 days, 1-μL aliquots were injected into an Agilent Technologies Inc. (Santa Clara, CA) 5975C inert XL gas chromatograph-mass spectrometer (GC–MS), fitted with an Rtx-5MS with Integra-guard (5% diphenyl/95% dimethyl polysiloxane) 30 m × 250 µm × 0.25 µm film thickness capillary column. The standard quadrupole GC–MS was operated in the electron impact (70 eV) ionization mode, targeting 2.5 full-spectrum (50–650 Da) scans per second, as described previously [55]. Metabolite peaks were extracted using a key selected ion, characteristic *m/z* fragment, rather than the total ion chromatogram, to minimize integrating co-eluting metabolites. The extracted peaks of known metabolites were scaled back up to the total ion current using predetermined scaling factors. Peaks were quantified by area integration and concentrations normalized to the quantity of the internal standard (sorbitol) recovered, amount of sample extracted, derivatized, and injected. A large user-created database (> 2400 spectra) of mass spectral electron impact ionization (EI) fragmentation patterns of TMS-derivatized compounds, as well as the Wiley Registry 10th Edition combined with NIST 2014 mass spectral database, were used to identify the metabolites of interest to be quantified. Unidentified metabolites were denoted by their retention time as well as key mass-to-charge (*m/z*) ratios and partial naming given the typical identity of specific *m/z*.

### Statistical analysis

Statistical analysis to determine statistical significance was performed by Student’s *t* tests of paired samples (against WV94). We used the *t* test function integrated in Excel software with *p* < 0.01 (Microsoft, Redmond, WA). The asterisk in each figure indicates significant difference compared to WV94 or control samples (*p* < 0.01 or < 0.05).

## Additional files

**Additional file 1.** Protein domain structure prediction of PtDUF231 family proteins.

**Additional file 2.** *PdDUF231A* expression in *OXPdDUF231A* transgenic *Populus.* RT-PCR was performed using cDNA generated from total RNA isolated from petiole of mature leaves. *PdUBCc* was used as an internal control. Red asterisks indicate the two transgenic lines selected for subsequent analyses.

**Additional file 3.** The gene copy number of *PdDUF231A* in *OXPdDUF231A* transgenic plants.

**Additional file 4.** Fold change and relative metabolite concentrations (µg/g FW sorbitol equivalents) of *OXPdDUF231A* transgenic plants versus empty vector control (K-147) *Populus deltoides* ‘WV94’ leaves (LPI 5) of 6-month-old greenhouse-grown plants. Metabolites were analyzed as trimethylsilyl derivatives by gas chromatography-mass spectrometry. Data are the average (Avg) and standard error of the mean (sem) of six *OXPdDUF231A* plants (three plants from each of two independent transgenic lines) and three K-147 control plants. Unknown and partially identified metabolites are designated by retention time, key mass-to-charge (*m/z*) ratios, and identified moieties.

**Additional file 5.** Primers used for RT-PCR and quantitative RT-PCR analyses in this study.

## Abbreviations

DUF231: Domain of Unknown Function 231
GPC: gel permeation chromatography
NMR: nuclear magnetic resonance
TM: transmembrane domain
