# Supplementary figures and images for: Overexpression of a Domain of Unknown Function 231-containing protein increases O-xylan acetylation and cellulose biosynthesis in Populus
Source: Biotechnol Biofuels. 2017 Dec 27;10:311. doi: 10.1186/s13068-017-0998-3 (PMC5744390; doi:10.1186/s13068-017-0998-3)

## Slide 1
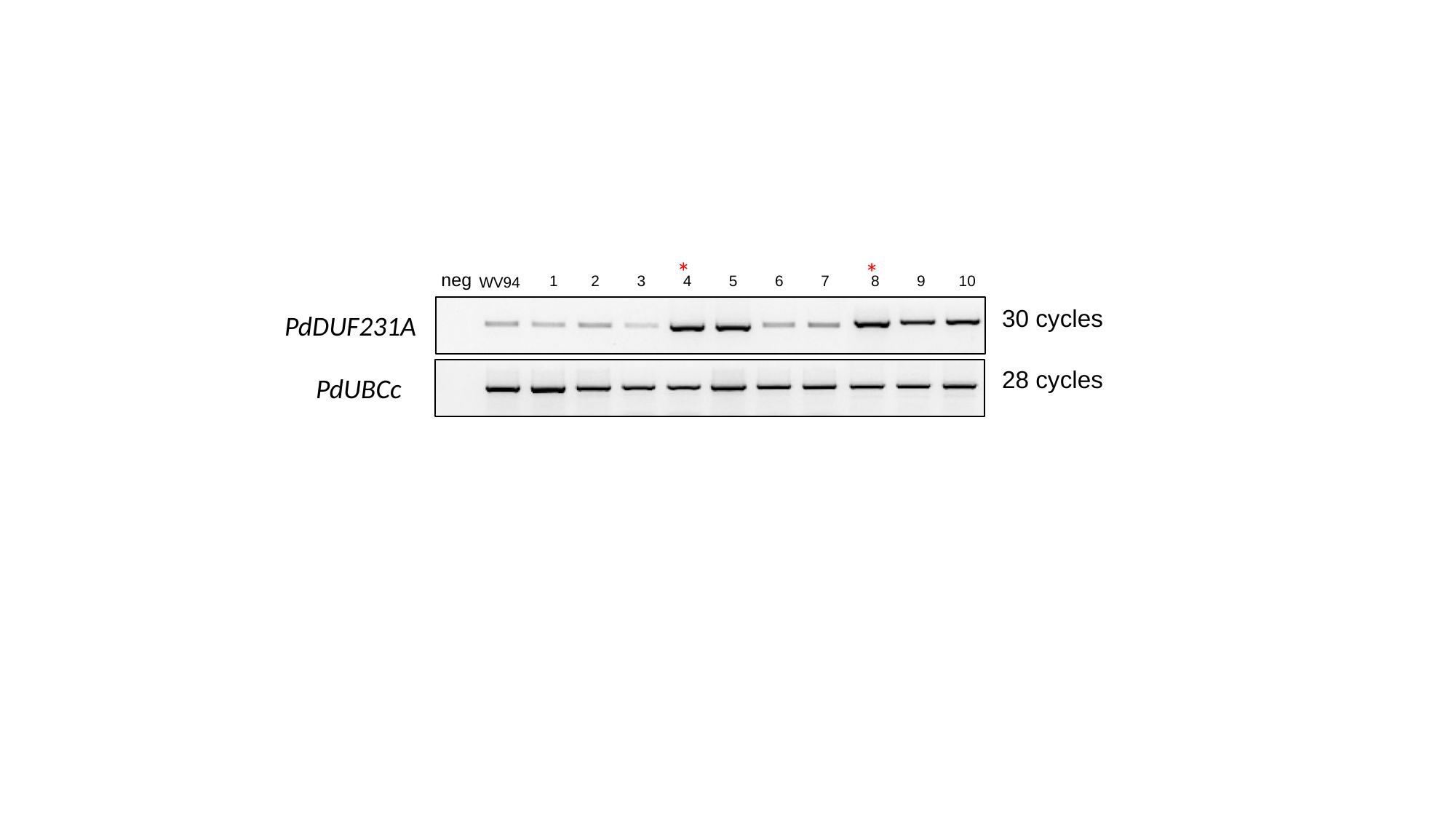

*
*
neg
1 2 3 4 5 6 7 8 9 10
WV94
30 cycles
PdDUF231A
28 cycles
PdUBCc

Supplement: Supplementary file 2 — Additional file 2. PdDUF231A expression in OXPdDUF231A transgenic Populus. RT-PCR was performed using cDNA generated from total RNA isolated from petiole of mature leaves. PdUBCc was used as an internal control. Red asterisks indicate the two transgenic lines selected for subsequent analyses. [file 13068_2017_998_MOESM2_ESM.pptx]

## Slide 1
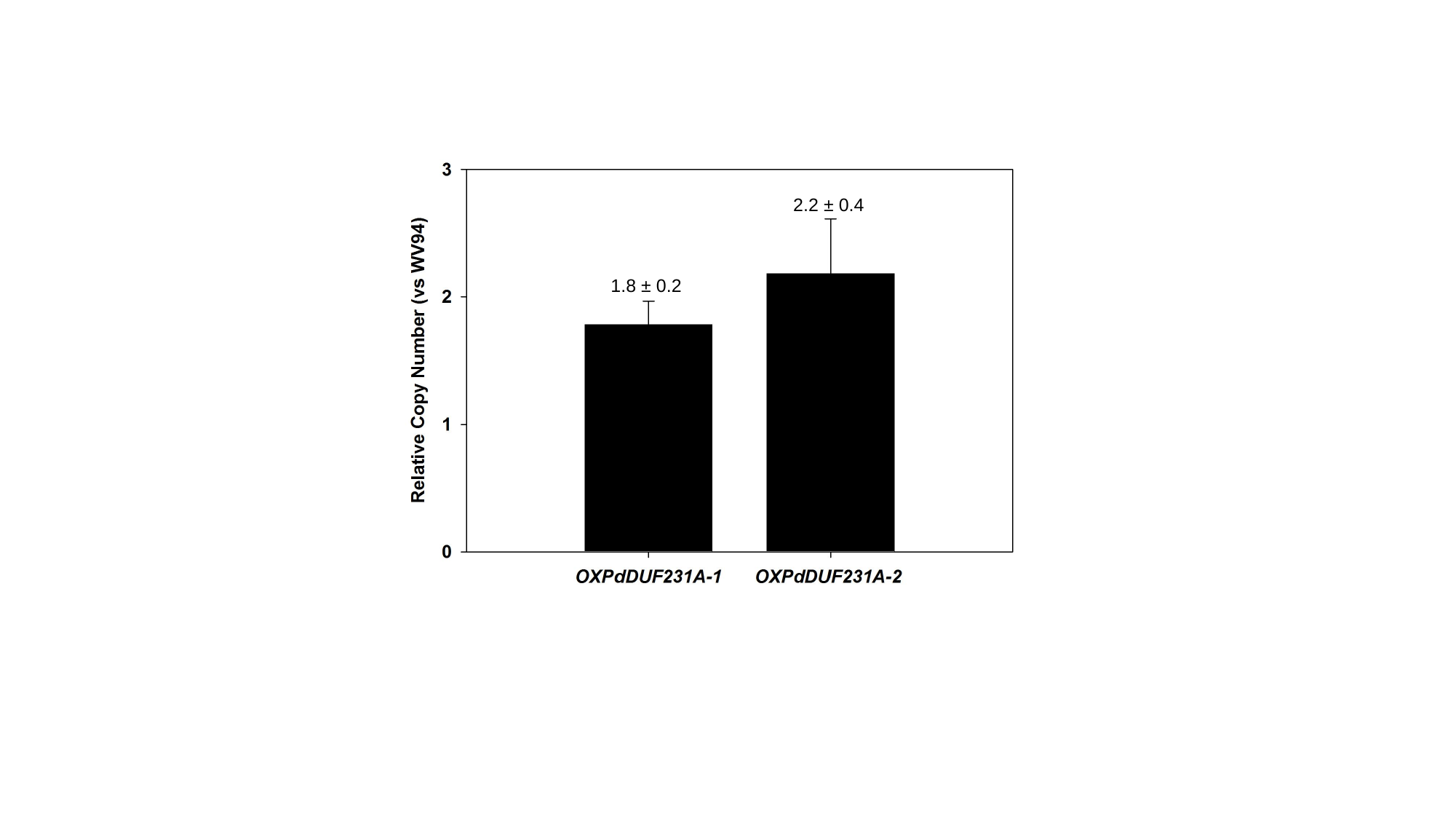

2.2 ± 0.4
1.8 ± 0.2

Supplement: Supplementary file 3 — Additional file 3. The gene copy number of PdDUF231A in OXPdDUF231A transgenic plants. [file 13068_2017_998_MOESM3_ESM.pptx]
